# Clinical trial of modulatory effects of oxytocin treatment on higher-order social cognition in autism spectrum disorder: a randomized, placebo-controlled, double-blind and crossover trial

**DOI:** 10.1186/s12888-016-1036-x

**Published:** 2016-09-21

**Authors:** Katrin Preckel, Philipp Kanske, Tania Singer, Frieder M Paulus, Sören Krach

**Affiliations:** 1 Max Planck Institute for Human Cognitive and Brain Sciences, Stephanstraße 1A, 04107 Leipzig, Germany; 2 Universitätsklinikum Schleswig-Holstein, Campus Lübeck, Klinik für Psychiatrie und Psychotherapie, Ratzeburger Allee 160, 23562 Lübeck, Germany

**Keywords:** Autism spectrum disorder, Oxytocin, Emotional reactivity, Social motivation, Social reward, Empathy, Theory of mind, fMRI, Oxytocin receptor gene

## Abstract

**Background:**

Autism spectrum disorders are neurodevelopmental conditions with severe impairments in social communication and interaction. Pioneering research suggests that oxytocin can improve motivation, cognition and attention to social cues in patients with autism spectrum disorder. The aim of this clinical trial is to characterize basic mechanisms of action of acute oxytocin treatment on neural levels and to relate these to changes in different levels of socio-affective and -cognitive functioning.

**Methods:**

This clinical study is a randomized, double-blind, cross-over, placebo-controlled, multicenter functional magnetic resonance imaging study with two arms. A sample of 102 male autism spectrum disorder patients, diagnosed with Infantile Autistic Disorder (F84.0 according to ICD-10), Asperger Syndrome (F84.5 according to ICD-10), or Atypical Autism (F84.1 according to ICD-10) will be recruited and will receive oxytocin and placebo nasal spray on two different days. Autism spectrum disorder patients will be randomized to determine who receives oxytocin on the first and who on the second visit. Healthy control participants will be recruited and case-control matched to the autism spectrum disorder patients. The primary outcome will be neural network activity, measured with functional magnetic resonance imaging while participants perform socio-affective and -cognitive tasks. Behavioral markers such as theory of mind accuracy ratings and response times will be assessed as secondary outcomes in addition to physiological measures such as skin conductance. Trait measures for alexithymia, interpersonal reactivity, and social anxiety will also be evaluated. Additionally, we will analyze the effect of oxytocin receptor gene variants and how these potentially influence the primary and secondary outcome measures. Functional magnetic resonance imaging assessments will take place at two time points which will be scheduled at least two weeks apart to ensure a sufficient wash-out time after oxytocin treatment. The study has been approved by an ethical review board and the competent authority.

**Discussion:**

Revealing the mechanisms of acute oxytocin administration, especially on the socio-affective and -cognitive domains at hand, will be a further step towards novel therapeutic interventions regarding autism.

**Trial registration:**

German Clinical Trial Register DRKS00010053. The trial was registered on the 8^th^ of April 2016

## Background

Autism Spectrum Disorders (ASD) are neurodevelopmental disorders characterized by deficits in social interaction and communication [1]. These include a broad variety of symptoms, ranging from a lack of interest in personal relationships to more subtle difficulties in managing complex social interactions. Neuroimaging studies on ASD were able to relate social impairments to specific dysfunctions in neural networks involved in processing social information such as the dorsal anterior cingulate (dACC), the precuneus, the anterior insula (AI) and the temporo-parietal junction (TPJ) [2, 3].

Recent literature suggests that oxytocin (OXT) plays a crucial role in social behavior. A single dose of intranasal OXT has been shown to increase in-group trust and fixation on the eye region, ease affect and face recognition, and improve empathy and Theory of Mind (ToM), pair bonding and affiliation, motivation, social cognition and social stress regulation in healthy participants, thus, a large range of social behaviors [4–9]. Based on emotional processing experiments, it has been suggested that amygdala reactivity mediates OXT effects on behavior [10, 11]. The amygdala is an oxytocin receptor (OXTR) rich area. OXT’s impact is, however, not limited to areas that possess OXTRs, because the areas that do, such as the amygdala, interact with other neurotransmitter systems, for example with dopamine and serotonin [5, 12]. These interactions may explain OXT findings in the prefrontal cortex (PFC) during altruistic interactions, as well as activation in the temporal lobe and the insula when emotions are processed [13]. The structure of a brain region and its activation patterns may also be influenced by genetic variations such as polymorphic variants in the promotor region of the OXTR gene, and by epigenetic mechanisms, such as methylation, which may in turn be related to individual differences in social behavior. Interestingly, hypermethylation of the OXTR gene, meaning less available OXTR mRNA, has been related to ASD and OXTR methylation influences social perception processes [14], which are impaired in ASD patients [15].

A recent meta-analysis, which included randomized clinical trials from January 1990 to September 2013 showed promising effects of OXT interventions on autistic traits, especially regarding emotion recognition and eye-gaze behavior [16]. The meta-analysis included seven independent studies with a total sample size of *N* = 95 male ASD patients. Significant decrease in repetitive behavior after OXT administration was found [17, 18], however not consistently [19]. Four studies investigated OXT effects on communication and social emotional processing. Two of these studies examined eye-gaze, one which reported an increased fixation pattern on the eye regions of presented stimuli after OXT administration [20], the other reporting no treatment effects on eye-gaze [19]. The remaining two studies evaluated OXT effects on emotion processing and revealed improved affective speech recognition after OXT administration [21] and increased performance in the Reading-the-Mind-in-the-Eyes task [22], which has been proposed to index ToM capacities [23]. However, no significant treatment-related improvement of emotion recognition was found in the University of New South Wales (UNSW) Facial Emotion Task [19]. ASD patients displayed increased sensitivity to complex social cues (i.e. unfair behavior) during a ball-tossing game and became more discriminative in their cooperative behavior, after receiving OXT [20]. Despite specific findings on OXT’s beneficial role on emotion recognition and quality of life measures [18], OXT did not consistently show improvements on the core symptoms of ASD.

Our own literature search (PubMed, Google Scholar, until May 30th 2016) revealed that since this initial meta-analysis on OXT effects in autism, eight further studies have been published that investigated OXT effects on ASD patients. Long-term OXT treatment reveals improvements in social reciprocity [24] and social interaction questionnaires [25] in ASD patients, while the findings of beneficial effects of acute OXT on social cognition are inconsistent [26, 27]. Implicit social processing was improved in ASD patients after acute and long-term OXT administration [24, 28]. Furthermore, salience of social stimuli increased significantly after long-term OXT administration [24] and reciprocal social interaction is associated with increased orbitofrontal cortex (OFC) activity after acute OXT administration [29].

Caregiver-rated questionnaires showed an improvement in social and developmental behavior of children with ASD, after long-term OXT administration [30]. In ASD patients, OXT increases trust in spite of preceding unfair treatment. These findings are accompanied by reduced amygdala, hippocampus and medial temporal gyrus activity compared to the PLC group [29]. Behavioral responses of ASD patients in a complex vicarious social pain task were not related to brain activity in the AI (as they were in healthy controls (HCs)), but rather to activity in the hippocampus [3]. OXT effects on empathy-evoking pictures did not significantly differ from those in the PLC group [31]. Cognitive and emotional empathy measured with physiological tools such as skin conductance responses (SCR) was equal in ASD patients and HCs, whereas mood ratings were significantly different between the groups [32]. It has previously been shown that ASD patients do not have a deficit in empathy per se, but that the deficit observed in individuals with an ASD diagnosis is rather related to increased alexithymia [33, 34]. Thus, patients do perceive negative affect as strong as HCs, but they are not able to translate this perception into a behavioral report of their affective state, when they are asked to state their mood and arousal [32].

Given these partially inconsistent results, the most recent review on OXT effects on ASD by Lee et al. [35] discusses whether the promising expectations of OXT as a potential treatment for ASD can still be maintained. The authors conclude that there is sufficient evidence for OXT to be a potential therapeutic for ASD and that further research should be carried out [35]. The same conclusion was reached by Meyer-Lindenberg and colleagues [5] who particularly emphasize the need for elaborate social cognition paradigms to determine potential modulatory OXT effects on corresponding neural networks [5].

The study at hand is part of a bigger autism research consortium - the ASD-Net, funded by the German Federal Ministry of Science (BMBF). The ASD-Net examines autism from many different angles, which allows each separate study to focus on very specific aspects in well characterized ASD populations. The present study is based on previous findings and satisfies the contemporary necessity of basic research to investigate the acute OXT effects on neural activity related to diverse social processes in ASD. The paradigms which are chosen for this study cover a wide range of socio-affective processes, raging from emotional reactivity to social reward, empathy and ToM.

### AIM

The primary aim with this clinical trial is to identify OXT’s acute mechanism of action on neural network activity and relate these to improvements in social cognitive skills in ASD patients. We will further investigate the differences between ASD patients and HCs. The results of this study may ultimately lead to the provision of care for social deficits that accompany ASD. Furthermore, a concomitant project in which the OXTR genotype will be determined may enable us to specifically identify individuals who benefit most from OXT treatment.

## Methods

### Design and settings

This study description is in accordance with the Consolidated Standards of Reporting Trials (CONSORT) guidelines that are published for the evaluation of randomized controlled trials [36].

### Trial design

**Fig. 1:**
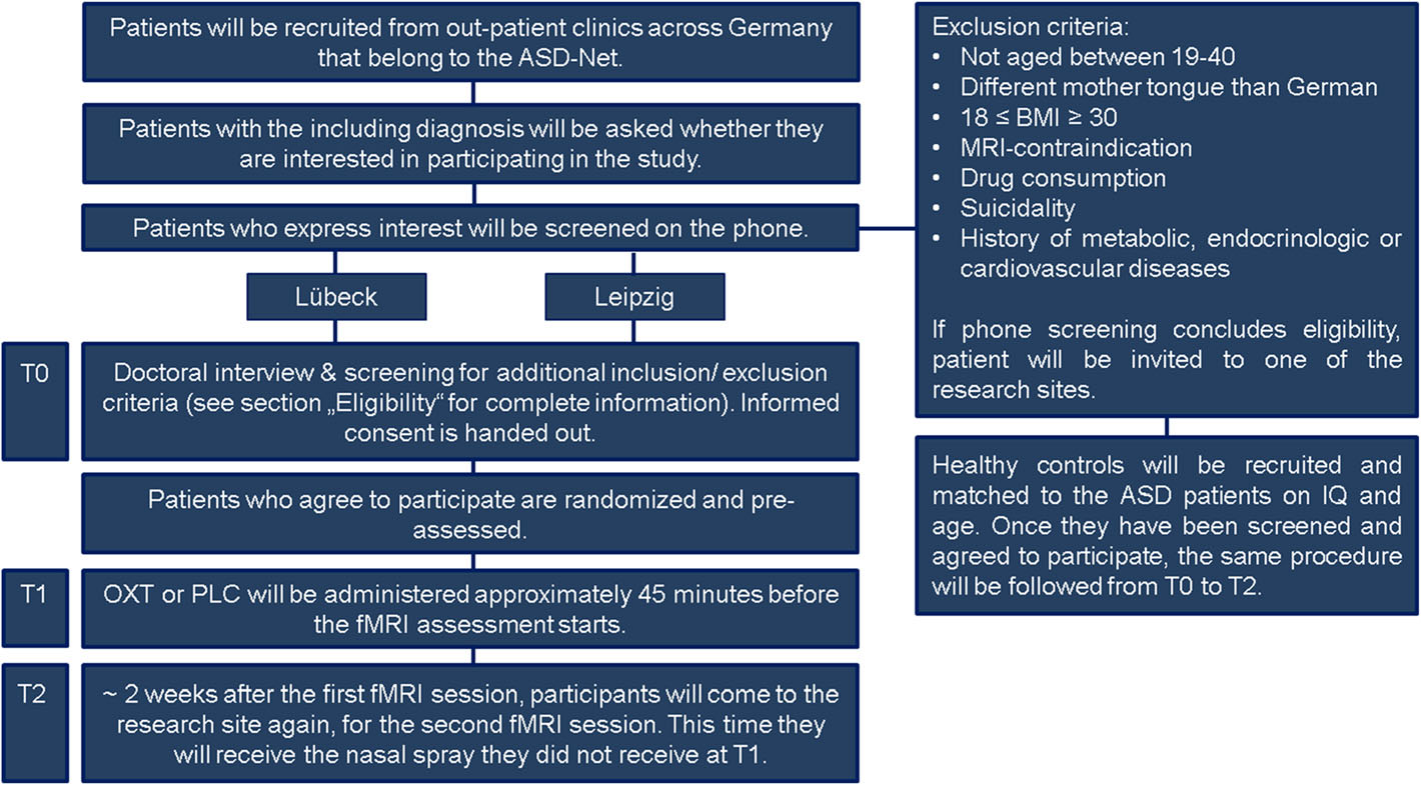
Study Design

This clinical trial is a multi-centered, two-arm, randomized, double blind placebo-controlled study with a cross-over design. Three visits and two functional magnetic resonance imaging (fMRI) assessments will take place for the participants of both groups (ASD and HC). The first visit includes a medical interview and an explanation of the informed consent; this visit determines whether the participant can be included in the study (T0). Once the participant is eligible to participate, he will be randomized to arm A or arm B, which determines whether he receives OXT or placebo (PLC) at the next visit. During the next visit (T1) the OXT or PLC administration and the first fMRI assessment will take place. The next visit (T2) will include the same measurements as T1 and will be scheduled approximately two weeks after T1, to ensure no carry-over effects if OXT had been administered at T1. After completion of T1 and T2 each participant (HCs and ASD patients) will guess which nasal spray they have received (Fig. 1).

### Ethical consideration

This clinical trial has been approved by the leading ethics committee of Lübeck University, the concomitant ethical board of Leipzig and the higher federal authority: Bundesinstitut für Arzneimittel und Medizinprodukte (BfArM) in Bonn. Approval of local ethics has been obtained in centers at which ASD patients will be recruited. The study will be conducted in compliance with the guidelines for Good Clinical Practice and the Declaration of Helsinki. Prior to testing participants' informed consent will be acquired. This trial is registered in the German Clinical Trial Register (DRKS00010053).

### Participants and procedure

Patients will be recruited from outpatient clinics across Germany. Recruitment will take place for both sites (Leipzig and Lübeck) simultaneously until the desired sample size is reached. Nurse practitioners at the clinics are asked to provide an envelope containing study information (and an entry form) to all ASD patients who are eligible for study participation based on information from their medical record. If interested in participation, the entry form will be completed and sent to the researchers, who will then contact the participant by phone to provide further study information and to address questions. Those willing to participate are asked to fill out a screening questionnaire. After receipt of the completed questionnaire, the researcher will contact the patient once more to discuss the screening outcome and to review all eligibility criteria. Afterwards the patient will be invited to the respective research site for T0. The informed consent form will be provided for the participant at least 24 h before the appointment T0 to allow for sufficient time to prepare questions already before T0. At T0 the study doctor will inform the patient about all risks and benefits of the study. After this consultation the participant will have sufficient time to decide whether he would like to participate in the study or not. If the participant agrees, the informed consent form will be signed and collected. A copy will be handed out to the participant. Healthy controls will be recruited via in-house databases until each patient has a suitable match. After contacting and controlling for HCs eligibility for the study, the same procedure as for the patients will be followed.

### Eligibility

ASD patients will be eligible participants if: 1) they are male, 2) they are aged between 19 and 40 years, 3) their mother tongue is German, 4) they have an IQ above 70, 5) they have one of the following diagnoses: Infantile Autism, (F84.0 according to ICD-10), Asperger Syndrome (F84.5 according to ICD-10), or Atypical Autism (F84.1 according to ICD-10), 6) they have given written informed consent. ASD patients are not eligible to participate if: 1) they have a BMI lower or equal to 18 or above or equal to 30, 2) they show MRI-contraindications such as having a pacemaker or being claustrophobic, 3) they have oxytocin contraindications such as arrhythmia, 4) they are suicidal, or 5) they have a history of metabolic, endocrinologic or cardiovascular diseases.

Healthy controls are matched to ASD patients regarding age, gender and IQ; additional inclusion criteria are: 1) no autistic traits (Autism Quotient (AQ) below 32. This questionnaire is validated as a screening instrument and allows the detection of autistic traits. The cut off score of 32 and higher complies with previous autism research [37]. 2) No first or second degree relatives with autism (participants will be asked for the occurrence of Autism within their family). Further exclusion criteria for HCs include: 3) a history of psychological or neurological illnesses and 4) current psychological or psychiatric treatment (participants will be asked to provide this information, before further consideration).

### Sample size

The sample size is calculated for the two primary outcomes: 1) the effects of the OXT treatment on the neural system functioning in the ASD population and 2) the comparison between the ASD and the HC group in the placebo condition. The first comparison for the power calculation is the cross-over comparison of the OXT effects on neural network activity in the ASD patient group, because the recruitment plan is determined by the ASD patient group. Meta-analytic estimates of intranasal OXT effects on the behavior of ASD patients indicated a medium effect size of approximately *d* = 0.60. We expect neural OXT effects to be bigger than behavioral OXT effects, because OXT has a stronger penetrance on the brain. Therefore, we adjusted the effect size for OXT’s influence on brain activity to *d* = 0.65. For all power calculations, a power of 80 % and a significance level of alpha = 0.001 was chosen and the analyses were carried out with PASS12. To obtain sufficient power for two-tailed t-test analyses in a cross-over design, *N* = 88 ASD patients need to be included in the analyses. Based on our prior experiences with ASD patients in behavioral and fMRI studies [3, 38] and the relatively short duration of the current study led to a drop-out estimation of 13 %. To allow for this drop-out, we need to initially recruit *N* = 102 patients. Due to the one on one matching it is necessary to include as many HCs as patients (*N* = 88). Recruitment for HCs will thus continue until each ASD patient has a proper match.

### Randomization

After completion of baseline assessment, ASD patients will be assigned to either arm A (T1: OXT, T2: PLC) or arm B (T1: PLC, T2: OXT). The order will be determined with a block randomization of 2 or 4 participants per block for arm A and arm B. The order of the three different experimental paradigms is also randomized with a fixed block size of six (123/132/213/231/312/321) so that each combination of assessment order is possible to ensure equivalent OXT levels for all paradigms. Thus, a second randomization list for the different paradigms, one for arm A and one for arm B, will ensure that each order of the experimental paradigms will be equally distributed in both arms.

The randomization lists will be independently generated for both sites (Leipzig/ Lübeck) to guarantee balancing for both sites. Thus, there will be three lists for each site, one arm-randomization list and two paradigm randomization lists. The lists are solely generated for the ASD population; HCs will be matched to the patients. The matching is also done by each center separately. Every matched participant will receive the same experimental order as the corresponding ASD patient.

### Processes and interventions

Before participants will be included in this study, they will come to one of the research sites (Leipzig or Lübeck) and will be carefully informed about the aim, contents, the duration and potential risks and benefits of this study. They will be informed that their participation is voluntary and that they can decide to no longer participate at any moment.

During the doctoral interview each participant will be examined to ensure that all inclusion and no exclusion criteria apply. The participant will especially be informed about not drinking excessive amounts of water before nasal spray administration, due to the potential anti-diuretic side effects of OXT that may lead to a water-intoxication.

If the participant decides to take part in the study and has no more open questions, after having had sufficient time to consider it, he can sign the informed consent and will be included in the study. Afterwards the participant will be asked to fill in questionnaires, which will take 1 – 2 h of time.

If inclusion/ exclusion criteria have not changed at the time of the second visit, the experimental procedure will be started. If participants are willing to participate in the concomitant genetic project, which determines their OXTR genotype, they will be asked to give a saliva sample. Pulse and blood pressure will be measured before and after nasal spray administration, to monitor OXT’s potential side effects and inclusion/ exclusion criteria. Physiological data, such as fixation patterns, pupil dilation, heart rate, respiration, and skin conductance patterns will also be acquired during the fMRI assessments.

Approximately 45 min before the fMRI-measurement starts, each participant receives a single dose (24 IU) of the nasal spray which he will self-administer. The participant is carefully instructed on how to administer the nasal spray. Each puff contains 2 IU (only Oxytocin nasal spray), thus in total 6 puffs need to be administered in each nostril to reach 24 IU.

Before the fMRI-measurements start, the eye-tracker will be calibrated and a habituation measurement of 5-7 min will take place. During this time, the participants will not have a specific task.

The procedure followed at T1 will be repeated at T2. After T2 the participant will be paid for his timely expenditures (10 Euros per hour) and thanked for participation.

### MRI assessment battery

#### Emotional matching

The emotional matching task was selected as a well-established fMRI task investigating emotion processing. In order to further optimize the task, we adapted the original emotional matching paradigm [39] by choosing more comparable control stimuli. Participants are presented with a total of 60 faces (20 positive, 20 neutral, 20 negative), 60 social scenes (20 positive, 20 neutral, 20 negative) and 60 non-social scenes (20 positive, 20 neutral, 20 negative). This stimulus set results in 9 conditions: positive faces, neutral faces, negative faces, positive social scenes, neutral social scenes, negative social scenes, positive non-social scenes, neutral non-social scenes and negative non-social scenes. Each presented picture consists of three single pictures arranged in a pyramidal disposal, in which the picture on the top is identical to one of the pictures below. Participants need to decide by button press whether the left or the right picture matches the one on top. Each stimulus picture will be presented for 4 s and 4 pictures of the same category (e.g. positive social scenes) constitute one block. The different category blocks will be presented in a randomized manner. This experiment consists of two parallel stimulus sets so that version A can be shown at one visit and version B can be shown at the other visit.

#### Social orienting, social reward anticipation, and consumption

As social rewards play a crucial part in almost all aspects of life we chose the social incentive delay (SID) paradigm [40–42] as a well-established tool to investigate its neural underpinnings. The paradigm is an adaption of the monetary incentive delay (MID) [43] (which will be also examined as a control condition) and has been shown to successfully elicit ventral striatal activity, also in patients with ASD [44]. In the MID/SID, a potential gain depends on the participant’s ability to hit a button in time whenever a target symbol appears on the screen. Distinct cues preceding the target inform the participant whether a reward can be won. Each trial will involve two phases: the anticipation phase and the consumption phase. During the anticipation phase, the cue will be presented for 250 ms signaling the type of potential reward, that is, wallets with money in the MID and smiling faces for SID or empty wallets and neutral faces respectively for no reward. Circles and squares indicate the reward type. After a delay period a target symbol will appear on the screen and a button has to be pushed within a set time window (adapted for individual reaction time assessed prior to the experiment). In the consumption phase, success will be acknowledged by presenting the picture of the reward. After each trial, participants will be shown a blank screen as a low-level baseline. The paradigm consists of two parallel stimulus sets for the SID and the MID which will either be shown at T1 or T2.

#### Empathy, compassion and theory of mind

The EmpaToM is a novel fMRI paradigm that investigates empathy and theory of mind (ToM), within one task [45, 46]. Participants will be presented with 40 short (~15 s) video clips of 10 actors, each recounting four putatively autobiographic episodes. These narrations are either neutral or emotionally negative and demand either theory of mind or factual reasoning. In total there are four conditions: neutral + non-ToM, neutral + ToM, emotional + non-ToM, neutral + non-ToM. Each video is followed by the question “How do you feel?” and participants are supposed to give an answer on a visual analogue scale ranging from negative – positive. Afterwards participants are asked “How much compassion do you feel?” and are again supposed to answer on a visual analogue scale ranging from none – very much. Before the experiment begins, participants are carefully instructed and an explanation of what is meant by the term *compassion* is given. Finally, participants will have to answer a multiple choice question that relates to the content of the video, and is either asking for information that requires ToM capacities or for information that requires factual reasoning. The video trials are presented in a randomized manner. This experiment consists of two stimulus sets so that version A can be shown at one visit and version B can be shown at the other visit.

#### Resting state brain activity

Resting state (rs)-fMRI identifies baseline brain activity of spontaneous blood-oxygen-level dependent (BOLD) fMRI signals and its analysis reveals changes in organization of large-scale functional networks, including oxytocinergic and related networks. Participants will be instructed to lie in the scanner with their eyes open and to try focusing on the presented fixation cross.

#### Structural brain markers

The structural measurements in this clinical trial will serve the comparison between ASD patients and HCs, not the comparison of the treatment groups (OXT vs. PLC).

##### Diffusion tensor imaging (DTI)

One DTI measurement will be acquired from each participant. Participants will be asked to lie particularly still during this measurement.

##### T1-weighted image

A three dimensional T1-weighted MR image will be obtained from all participants. All images will be examined and approved for inclusion by the study doctor at the respective institute.

### Primary outcome

Neural correlates of socio-affective and -cognitive tasks in ASD patients and HCs will be assessed after PLC and after OXT administration at two time points (cross-over design) using fMRI. For this study the primary outcome measure will be the neural network activity that is associated with the different social paradigms.

### Secondary outcomes measures and potential outcome mediators

Secondary outcomes entail behavioral measures that are collected during the fMRI sessions, including response times and ratings. Further, physiological measures that indicate affective arousal related to dACC and AI activity such as pupil dilation [see [47] for further details] heart rate and skin conductance responses are collected in the fMRI and fixation patterns and respiratory frequency will be recorded.

In addition to the secondary outcome variables, multidimensional aspects of autism and participants personality traits will be assessed with a questionnaire test battery, including: basic sociodemographic questionnaire, basic diagnosis and medication questionnaire (for patients only), number-symbol-test, digit-repetition test (part of the Hamburg-Wechsler-Intelligenztest für Erwachsene (HAWIE, since 2012 WAIS-IV)), brief symptom inventory (BSI-53) [48], WHO Disability Assessment Schedule [49], childhood trauma screener [50], clinical global impression scale (for patients only) [51], Trail Making Test A and B [52], vocabulary knowledge test (WST) [53], behavioral inhibitory scale (BIS) and behavioral approach scale (BAS) [54], Barratt impulsiveness scale [55], positive and negative affect schedule, interpersonal reactivity index (IRI) [56], Toronto alexithymia scale (TAS-20) [57], social interaction anxiety scale (SIAS) [58], Beck depression inventory (BDI-2) [59, 60], autism quotient (AQ) [37] state-trait-anxiety index – state (STAI-S) and state-trait-anxiety index – trait (STAI-T) [61], and an intelligence quotient (IQ) – test will be done. All data information will be entered in a database and are intended to be used for statistical analyses.

### Statistical analysis

#### Behavioral analyses plan

Raw data of all key variables will be tested for normality prior to analysis. Appropriate transformations (or non-parametric tests) will be conducted accordingly. Analyses will be done using repeated-measures analysis of variance (AN(C)OVA) and mixed models as primary designs. Difference scores will be calculated on every dependent variable for participant (ASD vs. HC) and treatment (OXT vs. PLC) groups. All measures will be compared between participant (ASD vs. HC) and treatment (OXT vs. PLC) groups and interaction effects (group x treatment) will be explored. Trait questionnaires may additionally be used as regressors when they can potentially explain behavioral differences between participant or treatment groups. After the T1 and the T2 visit, participants will be asked to guess whether they have received OXT or PLC. This information allows us to calculate a chi square test after unblinding, to check whether participants knew or did not know which nasal spray they received.

#### Task-related functional MRI analysis

All fMRI data will be preprocessed with SPM and afterwards statistical analysis will be carried out using the general linear model [62]. For all experiments, onset and duration of the block or event will be modeled. The respective regressors will be convolved with a canonical hemodynamic response function (HRF) and head motion will be accounted for by modeling the six motion parameters for each subject. If the head movement exceeds the usual tolerance of 3 mm, additional movement corrections will be carried out. Differences between participant (ASD vs. HC) and treatment (OXT vs. PLC) groups and interactions between participant and treatment groups on BOLD signal changes will be evaluated for each task (ANOVA and/ or t-tests). Whole brain and paradigm-specific region of interest (ROI) analysis, based on information from previous studies assessing these paradigms and meta-analyses of the investigated functions will be done. For the *emotional matching task*, two main effects (sociality and emotional valence) as well as the interaction effect (sociality x emotional valence) will be calculated. In the *social and monetary incentive delay task* two main effects (task and phase) will be calculated as well as the interaction effect (task x phase). The *EmpaToM* will include main effect contrasts of empathy and ToM. For this paradigm we may for example use the AI and the dACC as ROIs, because these regions have consistently been identified with empathy [45, 46]. Furthermore valence and compassion responses might be added as parametric modulators to further explain task-related brain activation. All task-related main effects and interactions will be calculated for participant (ASD vs. HC) and treatment (OXT vs. PLC) groups. Personality trait scores may also be added as regressors in the fMRI analysis to investigate secondary research questions and explain specific variance. All interaction effects will be followed up and more detailed investigations will be carried out. Additionally, we will extract the peak BOLD-fMRI activation levels from ROIs that have previously been reported in these paradigms or meta-analyses, and import the extracted values into SPSS or R for additional statistical calculations. Furthermore, we plan to perform connectivity analyses with paradigm-specific peak regions. The results of each fMRI paradigm will be analyzed and published separately, before the results of multiple paradigms will be combined to generate an overarching picture of OXT’s effects on social cognition/social functioning.

#### Resting-state functional MRI analysis

The rs-fMRI data will be preprocessed with SPM and afterwards statistical analysis will be performed. Nuisance covariates will be removed from the data including six head motion parameters. For functional connectivity calculation, seed regions will be defined based on the activation peaks observed in the task-related fMRI contrasts of the present study and in previous studies on related functions. We will perform voxel-wise correlation analyses to generate functional connectivity maps. T-tests will be used to extract those brain regions that show significant positive correlations with our peaks. The rs-fMRI measures will, after primary analysis, be contributed to a consortium, and multiple researchers may make use of the data and choose their analysis tool of preference.

#### Diffusion tensor imaging analysis

Analysis of DTI data will be performed using state-of the art techniques. The DTI data acquired in this study does not belong to our primary or secondary outcome measures. Exploratory analysis may be conducted after investigating the primary and secondary outcome measures. The main interest with these DTI data is to identify group differences between ASD patients and HCs. The DTI data will be contributed to a consortium, and multiple researchers may make use of the data and choose their analysis tool of preference.

#### T-1 weighted image analysis

A structural MPRAGE t1-weighted image will be analyzed and preprocessed with SPM. It will mainly be used as a reference image for the functional paradigms. Furthermore, the potential differences between groups will be analyzed.

All secondary outcome measures will be analyzed analogously.

## Discussion

The neural processes and the specificity of OXT effects on the socio-affective and –cognitive functioning are not fully understood. Intranasal OXT shows promising effects on the core symptoms of social cognition and affected brain systems in ASD. To date, there is no pharmacological treatment for the social deficits in ASD. Currently, three medication classes that aim at treating repetitive behavior (neuroleptics, [63–65], and SSRIs [66]), hyperactivity, impulsivity, disinhibition and inattention (psychostimulants [67]) are highly studied in ASD research. However, none of these pharmacological treatments challenges the social deficits of ASD. The social deficits are the focus of this clinical trial and we will evaluate whether OXT can improve these deficits in ASD patients by systematically investigating different levels of social behavior using various methods. Due to this multi-measure approach and the use of different paradigms, this study provides new insights in the functional mechanisms of OXT on the core symptoms of ASD patients.

The broad spectrum of ASD poses an additional challenge to develop adequate treatments for autism per se. We will concentrate on three diagnoses of ASD in this clinical trial: Infantile Autistic Disorder, Asperger Syndrome and Atypical Autism and only focus on male adults aged between 19 and 40 years. The within-subjects comparisons in the ASD group (OXT vs. PLC) and the comparisons to the healthy control group in both treatment states will enable us to identify treatment-related changes.

One aim of this clinical trial is to successfully predict, by using neurobiological, behavioral, environmental, and genetic information, whether intranasal OXT is a promising treatment option for patients with Infantile Autistic Disorder, Asperger Syndrome or Atypical Autism. A limitation that this clinical trial shares with many studies [7, 9, 68, 69] is that only male subjects will be tested and thus findings cannot be generalized to women. However, this limitation also increases the internal validity of this trial. Furthermore, as acute OXT effects will be investigated, no statements about longitudinal treatment effects can be drawn from this trial. The simple comparison of treatment responders with treatment non-responders would indicate regions involved in treatment-specific response, the possibility that patients would have responded to alternative treatment strategies remains. Acknowledging these limitations, we hope the study results will assist to inform future therapeutic intervention regarding groups with social deficits, especially ASD patients. Findings by Yatawara [30] described above support the assumption that OXT can have beneficial consequences regarding social capacities and general developmental behaviors in autistic children. These early beneficial results in autistic children might smooth the way for improved social skills in adulthood. The collaboration within this ASD-Net will allow to directly exchange findings and build upon each other’s results. As such, it offers high internal validity of each trial by using reproducible experimental set-ups and by integrating knowledge from related investigations.

## Abbreviations

ACC: Anterior cingulate
ADOS: Autism diagnostic observation schedule
AI: Anterior insula
aMCC: Anterior midcingulate cortex
ASD: Autism spectrum disorders
BAS: Behavioral approach scale
BDI-2: Beck’s depression inventory
BfArM: Bundesinstitut für Arzneimittel und Medizinprodukte
BIS: Behavioral inhibitory scale
BOLD: Blood-oxygen-level dependent
BSI-53: Brief symptom inventory
dACC: Dorsal anterior cingulate
dmPFC: Dorsomedial prefrontal cortex
DTI: Diffusion tensor imaging
FA: Fractional anisotropy
fMRI: Functional magnetic resonance imaging
HAWIE: Hamburg-Wechsler-Intelligenztest für Erwachsene
HCs: Healthy controls
HRF: Hemodynamic response function
IFG: Inferior frontal gyrus
IQ: Intelligence quotient
IRI: Interpersonal reactivity index
MD: Mean diffusivity
NAcc: Nucleus accumbens
OFC: Orbitofrontal cortex
OXT: Oxytocin
OXTR: Oxytocin receptor
PFC: Prefrontal cortex
PLC: Placebo
rs: Resting state
SCR: Skin conductance responses
SIAS: Social interaction anxiety scale
SMG: Supramarginal gyrus
sMRI: Structural MR image
SSRIs: Selective serotonin reuptake inhibitors
STAI-S: State-trait-anxiety index – state
STAI-T: State-trait-anxiety index – trait
STS: Superior temporal sulcus
TAS-20: Toronto alexithymia scale
ToM: Theory of mind
TPJ: Temporo-parietal junction
UNSW: University of New South Wales
WST: Vocabulary knowledge test
ZKS: Zentrum für Klinische Studien
